# Effects of Home-Based Daily Respiratory Muscle Training on Swallowing Outcomes in Patients with Chronic Stroke: Protocol for a Randomized Controlled Trial

**DOI:** 10.3390/jcm13185547

**Published:** 2024-09-19

**Authors:** Mónica Zapata-Soria, Irene Cabrera-Martos, Alejandro Heredia-Ciuró, Esther Prados-Román, Javier Martín-Nuñez, Marie Carmen Valenza

**Affiliations:** Department of Physiotherapy, Faculty of Health Sciences, University of Granada, 60 Av. Illustration, 18016 Granada, Spain; monicazapatas@gmail.com (M.Z.-S.); ahc@ugr.es (A.H.-C.); espra66@hotmail.com (E.P.-R.); javimn@ugr.es (J.M.-N.); cvalenza@ugr.es (M.C.V.)

**Keywords:** respiratory muscles, stroke, deglutition disorders

## Abstract

(1) **Background:** Swallowing disorders are common following a stroke. This study aims to evaluate the effects of a home-based daily intervention focused on inspiratory and expiratory muscle training on swallowing outcomes in patients with chronic stroke. (2) **Methods:** This manuscript presents the protocol of a single-blind randomized clinical trial. Patients with chronic stroke will be randomly assigned to either an experimental or a control group. The experimental group will undergo daily home-based respiratory muscle training in addition to standard speech and language therapy, while the control group will receive only the standard intervention. The main outcome measures will include the aspiration risk, the strength of respiratory muscles, and peak cough flow. (3) **Results:** It is hypothesized that patients receiving home-based respiratory training in addition to standard therapy will achieve significant improvements in aspiration risk, respiratory muscle strength, and cough efficacy in comparison with those included in the control group. The results will be published as a manuscript. (4) **Conclusions:** This study aims to provide evidence on the effectiveness of home-based respiratory muscle training in enhancing swallowing function and respiratory parameters in patients with chronic stroke.

## 1. Introduction

Stroke is the second leading cause of death and the third leading cause of disability worldwide [1]. Among the most frequently reported symptoms, dysphagia occurs in up to 80% of stroke survivors, with varying progression and severity [2,3]. A study conducted by Arnold et al. [4] observed that more than one in five patients with ischemic stroke suffer from swallowing dysfunction. Vasan et al. [5] also showed that approximately 10% of patients with intracerebral hemorrhage develop post-stroke dysphagia.

This dysfunction can result in social difficulties, reduced quality of life, malnutrition, dehydration, aspiration pneumonia, pulmonary complications, and recurrent coughing [2,6,7,8]. Moreover, it has been recognized as an independent risk factor linked to prolonged hospital stay, institutionalization after discharge, poorer functional recovery, and higher mortality rates. Therefore, effective management of this condition is key not only for stroke patients but also for the healthcare system [5,9].

Determining the optimal intervention for individual patients is a challenge that involves interdisciplinary work to prevent negative consequences and help the patients acquire functional swallowing [10]. Several techniques and exercises to improve swallowing ability and muscle function have been previously described. The main approach for managing swallowing dysfunction includes adapting food and fluid textures, postural techniques, compensatory techniques, and rehabilitation exercises [2,11,12,13,14,15,16]. These strategies include resistance, neuromotor, stretching, oral and lingual exercises, and myofunctional therapy [17]. Specifically, isotonic, and isometric tongue exercises, as well as lip force training have demonstrated improvement in the physiological function of oropharyngeal and submental muscles [18,19,20]. In addition, suprahyoid and infrahyoid exercises such as chin tuck against resistance (CTAR), and submandibular push have been shown to improve swallowing safety in patients with stroke [21,22]. 

Recent systematic reviews have explored the effects of respiratory training on swallowing disorders in patients with stroke, showing positive results on swallowing function and a reduction in complications such as pneumonia [23,24]. In respiratory training, patients are asked to perform repetitive breathing exercises using pressure threshold devices that provide different levels of flow-dependent resistance as external loads [25]. Respiration and swallowing share common anatomical structures and require adequate temporal coordination to ensure proper nutrition and prevent pulmonary aspiration and its sequelae [26]. Respiratory muscle weakness, a known risk factor for aspiration pneumonia, increases the risk of death by 8.5 times in individuals with chronic stroke who suffer from dysphagia compared to those without this condition [4].

Respiratory muscle training, including exercises targeting the inspiratory muscles, may increase muscle strength and improve muscle function, which is beneficial for achieving maximum lung volume at the beginning of a cough. Expiratory muscle strength training enhances the movement of the neurologically innervated submental muscle complex and increases intrathoracic pressure during coughing [16]. Although the results are promising, the effects of respiratory muscle training require further research using objective tools to quantify physiological effectiveness and better individualize training programs for patients with stroke and dysphagia. Previous studies have reported heterogeneity in the devices used, frequency, resistance, and progression of training. Moreover, several studies included in previous systematic reviews failed to provide data on these parameters and progression criteria [24,25]. 

Additionally, there is a need for studies on patients with chronic stroke, as most randomized controlled trials exploring the effects of respiratory muscle training on swallowing disorders in patients with stroke have focused on the acute and subacute stages of the disease. Although early dysphagia assessment is crucial for patient recovery [2,27], some symptoms and swallowing difficulties can persist in the long term [28]. Mao et al. [29]. identified a prevalence of 43.7% for chronic dysphagia in patients with ischemic stroke. Given the need for long-term care, rehabilitation, and community-based rehabilitation, clinical services focused on swallowing disorders are lacking [30,31]. The review conducted by Chen et al. [32] suggests that new therapy approaches, such as home-based stroke rehabilitation, could offer high-quality therapy and improve patients’ quality of life. This modality could increase the rehabilitation intensity to meet therapeutic needs without raising costs. No previous study has assessed the effects of home-based daily respiratory muscle training combined with standard therapy in patients with chronic stroke. Therefore, the objective of this study will be to evaluate the impact of a home-based daily intervention focused on inspiratory and expiratory muscle training on swallowing outcomes in patients with chronic stroke. 

## 2. Materials and Methods

### 2.1. Study Design

The research design will be a randomized, single-blind clinical trial. This study will analyze the effect of home-based daily respiratory muscle training in patients with chronic stroke presenting with swallowing dysfunction. This group will be compared with a control group. The timeline of the study is shown in Table 1, including the time point at which the enrollment and randomization procedure, assessments, and intervention will be conducted. 

### 2.2. Ethical Considerations

Written informed consent will be obtained from all the participants before enrollment in the study. In addition, the intervention was reviewed and approved by the Ethics Committee of Andalucía and conforms to the Helsinki Declaration. The study protocol has been registered at clinicaltrials.gov under the ID NCT06312319.

### 2.3. Setting 

The patients included in the study will conduct the intervention in their homes. Participants will be recruited from the Asociación Granadina de Familias por la Rehabilitación del Daño Cerebral Adquirido (AGREDACE) and the Asociación de Familiares y Enfermos de Ictus de Granada (Neuro-Afeic).

### 2.4. Participants

This study will include patients with a clinical diagnosis of stroke and dysphagia. Inclusion criteria will be as follows: age of 18 years old or over, and chronic stroke, defined as an open-ended period of ≥6 months’ duration since stroke diagnosis [33]. The exclusion criteria will include a clinical diagnosis of a concomitant neurological condition, co-occurring aphasia, severe cognitive impairment (Montreal Cognitive Assessment [MoCA] < 26 points) [34], tracheostomy, cancer, lack the neuromotor ability to perform respiratory function tests, central apnea, obesity-hypoventilation syndrome, or significant cardiorespiratory conditions such as hemodynamic instability, pulmonary embolism, recent pneumothorax, acute hemoptysis, active respiratory infections, recent myocardial infarction, unstable angina, pulmonary hypertension, uncontrolled asthma, or severe chronic obstructive pulmonary disease. Additionally, patients who have recently undergone otorhinolaryngological, abdominal, or thoracic surgery will be excluded.

### 2.5. Randomization

Participants in this study will be randomly assigned (1:1 ratio) to either an experimental or a control group. The eligibility of participants will be assessed by an independent researcher not involved in the randomization process. The randomization will be performed by a researcher not involved in the study procedure. The randomization sequence will be generated by a computer program (https://www.randomizer.org). The assessment will be carried out by a therapist blinded to the treatment assignments.

### 2.6. Intervention Protocol

**Figure 1:**
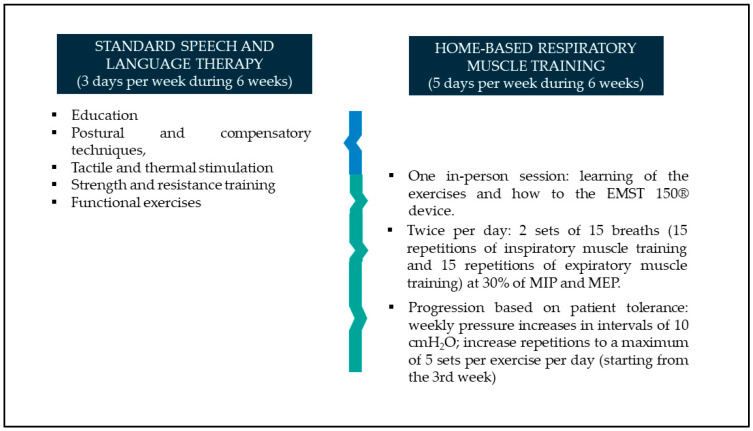
Protocol of the home-based respiratory muscle training and standard speech and language interventions.

Patients allocated to the experimental group will participate in a 6-week home-based respiratory muscle training program in addition to the standard speech and language intervention. Patients in the control group will receive only the standard intervention. The protocol of both interventions is illustrated in Figure 1. 

#### 2.6.1. Respiratory Muscle Training

Respiratory muscle training will be performed using a calibrated Expiratory Muscle Strength Trainer (EMST-150) and an inspiratory adapter (IA-150) (EMST 150; Aspire Products, Gainesville, FL, USA). 

In a previous in-person session, patients will be trained to use the device. They will be instructed to sit in a proper position in a chair, wear nose clips, take a deep breath, hold their cheeks lightly, blow as hard as they can into the device, and ensure that air flows freely through the device. Written instructions with pictures and text will also be provided to each patient.

The home-based respiratory training sessions will consist of two sets of 15 breaths (15 repetitions of inspiratory muscle training and 15 repetitions of expiratory muscle training) with 2 min of recovery between the sets. Training will be conducted twice daily, 5 days per week for 6 weeks, at the same time each day. Training loads will be set at a pressure equivalent to 30% of maximal inspiratory and expiratory pressures and will be increased weekly at intervals of 10 cmH_2_O [35,36]. This resistance will be readjusted if needed according to the patient’s tolerance. The number of repetitions will also increase starting from the third week to a maximum of 5 sets of each inspiratory and expiratory exercise per day, as tolerated. Patients will complete a diary indicating the number of repetitions per day and the time of day. Additionally, patients will use a visual analog scale from 0 to 10 to rate their symptoms of fatigue, dizziness, or pain during the training, where 0 represents no symptoms and 10 represents maximal severity. Once per week, the patient will have a video call from the therapist to review the diary and any symptoms that appeared during training, adjust the progression, and resolve any doubts. Adherence will be assessed using this diary. 

#### 2.6.2. Standard Speech and Language Therapy

All patients will receive standard speech and language interventions for one hour per day, 3 days per week for 6 weeks. The intervention will include (1) education focused on dysphagia and airway protection, including how to modify food and fluid textures; (2) postural and compensatory techniques; (3) tactile and thermal stimulation with different temperatures, flavors, and textures; (4) strength and resistance training including isotonic and isometric exercises such as CTAR or submandibular push; and (5) functional exercises based on videofluoroscopic findings [37]. The frequency and intensity of the exercises will be individualized, taking into account the safety level and the types of food that patients can eat safely, with modifications to texture if necessary. Attendance will be registered weekly.

### 2.7. Outcomes

At baseline, clinical and demographic data will be recorded, including age, sex, height, weight, academic studies, the side, site, and the dimension of the cerebral lesion based on the Fugl-Meyer Upper and Lower Extremity scales [38]. Additionally, disease duration and the type of stroke, whether ischemic or hemorrhagic, will be noted. In cases of ischemic stroke, the type of recanalization therapy will be specified. The initial evaluation will be performed once informed consent is obtained. The assessment will be conducted by a blinded therapist with previous experience in treating patients with stroke. 

#### 2.7.1. Main Outcomes

The main outcomes will include the aspiration risk, the strength of respiratory muscles, and peak cough flow. 

The penetration or aspiration risk will be assessed using the Penetration–Aspiration Scale (PAS) [39]. The PAS is a valid and reliable 8-point scale used during videofluoroscopy assessment. It evaluates the depth of airway invasion, the material remaining after swallowing, and the patient’s response to aspiration. The scores range from 1 (material does not enter the airway) to 8 (material enters the airway, passes below the level of the vocal folds, and no effort is made to eject it). The assessment will be conducted by two experienced researchers, who are blinded to the swallowing performance before and/or after the targeted swallow task. This scale has been reported to be a valid tool for differentiating between normal and abnormal airway protection [39]. 

Respiratory muscle strength will be evaluated using maximal inspiratory pressure (MIP) and maximal expiratory pressure (MEP) measurements [40]. The patients will be evaluated in a sitting position while wearing a nose clip. To evaluate the MIP, the patients will be instructed to exhale air near the residual volume and then inhale through the mouthpiece. To measure MEP, the patients will be asked to inhale air near total lung capacity and then exhale the air through the mouthpiece. Three maneuvers will be performed, ensuring no more than a 20% difference among them [41]. The cut-off values to determine muscle weakness are 62 and 83 cmH_2_O for MIP in females and males, respectively, and 81 and 109 cmH_2_O for MEP in females and males, respectively [42].

Voluntary peak cough flow (L/min) will be evaluated using a peak flow meter (Micro Medical, Cambridge, UK) [43]. This parameter assesses the maximum expiratory flow during a cough to evaluate the efficacy of secretion clearance. The patient will be seated in a chair, will inhale as much air as possible, and then cough as forcefully as possible through the mouthpiece. The maneuver will be performed three times, and the mean of the values will be included in the analysis. Standardized values in healthy adults range between 360 and 400 L/min [44]. An effective cough is associated with having a peak flow rate of over 160 L/min, while a rate below 270 L/min is associated with increased secretion retention and a higher risk of infection [45].

#### 2.7.2. Secondary Outcomes

Secondary outcomes will include the functional ability of oral intake and the quality of life related to dysphagia.

The Functional Oral Intake Scale (FOIS) is used to measure a patient’s level of eating ability. The scale ranges from 1 (no ability to swallow) to 7 (normal swallowing). Scores from 1 to 3 correspond to non-oral feeding or requiring additional tube feeding, while scores from 4 to 7 correspond to an oral diet, considering special modifications and compensations at certain levels. The FOIS is a valid and reliable scale for stroke patients [46].

The quality of life of patients with dysphagia will be assessed using the Swallowing Quality of Life questionnaire (SWAL-QOL) [47]. This tool is composed of 44 items divided into 11 subscales. The assessment items include burden, food selection, eating loss, eating duration, eating desire, fear, sleep, fatigue, communication, mental health, and social functioning. Each item is scored according to a Likert scale ranging from 1 (worst condition) to 5 (best condition). Lower scores are associated with poorer quality of life. SWAL-QOL is a valid and reliable scale for patients with dysphagia.

### 2.8. Sample Size Calculation

The sample size was calculated using G*Power software, version 3.1.9.2. Based on a previous study, our control group’s expected post-treatment PAS score is 4.62 ± 0.77 [48]. According to a power calculation of 80% and assuming a clinically relevant difference of one point on the PAS score between the study groups, with a standard deviation of 1.2 [49], we determined a total sample size of 55 participants. The calculation is based on an alpha level of 95%, accounting for a 15% dropout rate.

### 2.9. Statistical Analysis

Data will be analyzed using the Statistical Package for the Social Sciences (SPSS) for Windows (version 26, IBM, Armonk, NY, USA). Categorical data will be reported as frequencies and percentages, while continuous variables will be summarized using means and standard deviations. The Shapiro–Wilk test will be used to assess data normality. Differences between groups will be examined with the Chi-square test for categorical variables. For continuous variables, Student’s t-test will be applied for normally distributed data, and the Mann–Whitney U test will be used for non-parametric data. Effect sizes will be calculated using Cohen’s d, with the following values: a small effect is indicated by 0.3, a moderate effect is indicated by 0.5, and a large effect is indicated by 0.8.

Statistical significance will be determined at *p* < 0.05. Participants with adherence rates of 80% or less, based on home diaries and attendance to standard speech and language sessions, will be excluded from the statistical analysis.

## 3. Results

The expected outcomes include improved swallowing function. Patients are supposed to improve swallowing safety and efficiency. The addition of respiratory training to standard intervention is expected to improve coordination between breathing and swallowing, thereby reducing the risk of aspiration and facilitating better management of various food textures. A reduction in the frequency and severity of aspiration events is expected, leading to improved airway protection and a lower incidence of aspiration pneumonia. The respiratory training program is designed to strengthen both inspiratory and expiratory muscles. As a result, significant gains in respiratory muscle strength are expected, as evidenced by increased MIP and MEP values. Patients are also expected to achieve a more effective cough, which is relevant for clearing the airway and reducing the risk of aspiration pneumonia. This improvement should be reflected in higher peak cough flow measurements. Enhancements in swallowing ability are likely to contribute to a better quality of life, including reduced symptoms related to dysphagia and increased comfort during eating and drinking.

The home-based format of the program is expected to improve adherence, promote consistent participation, and achieve better long-term results. By integrating respiratory training with standard therapeutic interventions, the program aims to address both swallowing and respiratory issues more effectively, leading to improved overall rehabilitation outcomes.

The authors will present the study results in a peer-reviewed journal. The procedures and patient involvement at various stages of the study will be illustrated in a flow diagram. The article will highlight the main findings, starting with a table that details the descriptive characteristics of the sample. This will be followed by data comparing baseline outcomes between groups for both primary and secondary measures. Finally, the results of the intervention outcomes will be presented, including within-group and between-group changes, with effect sizes calculated using Cohen’s d.

## 4. Discussion

This study aims to evaluate the impact of a home-based daily intervention focused on inspiratory and expiratory muscle training on swallowing outcomes in patients with chronic stroke. It is hypothesized that patients receiving home-based respiratory training in addition to standard therapy will achieve significant improvements in aspiration risk, respiratory muscle strength, and cough efficacy.

Post-stroke dysphagia can cause health-related problems throughout a patient’s life and is linked to increased healthcare costs [50]. In this context, this study aims to increase the intensity of therapy by using a home-based approach in addition to standard intervention. Implementing home-based interventions allows patients to reduce the frequency of hospital visits. Additionally, with continuous monitoring and personalized care plans, patients with dysphagia often experience better health outcomes and a reduced need for emergency interventions related to respiratory complications [32,50,51].

Previous studies [52,53] have assessed the effects of home-based exercises for patients with dysphagia. Yang et al. [52] conducted a systematic review of home-based telerehabilitation for dysphagia in patients with head and neck cancer. They observed improvements in swallowing safety, adherence to rehabilitation, and quality of life related to swallowing dysfunction. Moreover, the home-based approach demonstrated good efficiency and low costs compared with face-to-face interventions [52]. Additionally, a study involving respiratory muscle training was conducted in patients with Parkinson’s disease presenting with dysphagia using a home-based approach [46]. The authors reported that four-week expiratory muscle strength training significantly reduced dysphagia severity, with a sustained effect after 3 months compared to sham training [53]. In this protocol, we present a home-based approach based on respiratory training for patients with chronic stroke and dysphagia.

Respiratory muscle training has previously shown improvement in respiratory muscle strength, pulmonary function, and functional capacity in early stroke [23,24]. The effects of respiratory muscle training focus on improving swallowing mechanics, which allows for the strengthening of the respiratory muscles and helps generate a more efficient cough, essential for reducing the risk of aspiration pneumonia. Voluntary coughing protects the airway and clears aspiration from the subglottic area [54,55]. Additionally, coordination between breathing and swallowing may be enhanced, minimizing the risk of aspiration during the swallowing-respiration cycle. However, due to the heterogeneity of subjects with dysphagia, the optimal dosage and intensity of exercises for swallowing disorders remain unclear, necessitating further studies to determine these parameters [17].

The studies focused on respiratory training for dysphagia have primarily targeted acute or subacute stroke; more studies on chronic stroke are needed [23,24]. Some authors have reported that swallowing dysfunction persists in patients with chronic stroke [28,29]. However, the improvement of respiratory muscle strength in chronic stroke is not well understood due to the limited studies at this stage of stroke [23,24]. This study focuses on patients with chronic stroke and dysphagia. Combined respiratory training protocols could enhance overall swallowing safety and efficiency in dysphagic post-stroke patients [23,24]. The results of our study will also provide evidence regarding the effects of respiratory intervention in chronic stroke when added to standard intervention. The daily intervention will allow us to increase the intensity of the treatment, overcoming barriers such as therapist or patient availability, extra intervention costs, and patient compliance in the chronic stroke stage.

A study conducted by Krekeler et al. [17] observed that the dosage and intensity in clinical practice are lower compared to research studies. In our study, the respiratory muscle training devices are relatively inexpensive, portable, and easy to use. They require no specialist assistance, making them suitable for use in a home environment. A previous trial focused on supervised, home-based respiratory muscle training showed positive results, achieving a significant improvement in the quality of life of the participants [56]. Our study seeks to increase accessibility to therapy and improve the benefits of the standard intervention by increasing the dosage. Treatment adherence is an important but still unresolved health issue, especially in interventions involving home-based exercises [57]. Increased compliance is linked to an increase in cost-effectiveness. Several systematic reviews have reported that diaries are one of the most used tools to measure adherence in home-based approaches [58,59]. In our study, the patients will complete a diary indicating the repetitions and the symptoms associated with exercise performance.

The proposed home-based respiratory training ensures an individualized program with the appropriate intensity and duration, adapted to the symptoms and baseline values of each participant. Adherence will be facilitated through regular monitoring and program adjustments based on patient progress and feedback. Additionally, the home-based respiratory training will be integrated with other therapeutic interventions for dysphagia, such as dietary modifications, postural adjustments, and traditional swallowing exercises, to achieve comprehensive rehabilitation.

Respiratory training has important clinical applications for managing post-stroke dysphagia. By strengthening both inspiratory and expiratory muscles, this training will improve the coordination between breathing and swallowing, frequently impaired in chronic stroke. Enhanced respiratory muscle strength supports a more effective cough, essential for clearing the airway and preventing aspiration. Furthermore, respiratory training aids in reestablishing better control over the swallowing–respiration cycle, thereby reducing the risk of aspiration pneumonia, a leading cause of morbidity and mortality among stroke survivors. The implementation of respiratory exercises into rehabilitation programs provides a non-invasive, cost-effective approach that can be conducted at home, improving patient adherence and increasing the overall intensity of therapy. This program is expected to improve not only swallowing safety and efficiency but also the quality of life of patients with chronic stroke and dysphagia.

## 5. Conclusions

This study aims to evaluate the effects of home-based daily intervention that focuses on inspiratory and expiratory muscle training on swallowing outcomes in patients with chronic stroke. The results are expected to offer a cost-effective approach, improving accessibility to rehabilitation.

## Figures and Tables

**Table 1:** Timeline of the study.

	Enrollment and Randomization	Pre-Intervention	Intervention (6 Weeks)	Post-Intervention
Timepoint	First month	Second month	Second and Third month	Fourth month
Enrollment				
▪ Recruitment of patients and verification of inclusion/exclusion criteria.	✓			
▪ Informed consent	✓			
▪ Allocation	✓			
Assessments				
▪ Aspiration risk		✓		✓
▪ Respiratory muscle strength		✓		✓
▪ Voluntary peak cough flow		✓		✓
▪ Functional oral intake level		✓		✓
▪ Quality of life		✓		✓
Interventions				
▪ Experimental Group: Home-based respiratory muscle training + standard speech and language therapy			✓	
▪ Control Group: Standard speech and language therapy			✓	

## Data Availability

Not applicable.
